# Determination of Impact Damage in CFRP via PVDF Signal Analysis with Support Vector Machine

**DOI:** 10.3390/ma13225207

**Published:** 2020-11-18

**Authors:** Hyun-Taik Oh, Jong-Ick Won, Sung-Choong Woo, Tae-Won Kim

**Affiliations:** 1 Department of Automotive Engineering, Hanyang University, Seoul 04763, Korea; oht0709@hanyang.ac.kr (H.-T.O.); jas1995@hanyang.ac.kr (J.-I.W.); 2 Researcher Center for Next Defense Convergence, Hanyang University, Seoul 04763, Korea; lilsuzy@hanyang.ac.kr; 3 Department of Mechanical Engineering, Hanyang University, Seoul 04763, Korea

**Keywords:** poly(vinylidene fluoride), impact damage, delamination, discrete wavelet transform (DWT), support vector machine (SVM)

## Abstract

Carbon fiber reinforced plastics (CFRPs) have high specific stiffness and strength, but they are vulnerable to transverse loading, especially low-velocity impact loadings. The impact damage may cause serious strength reduction in CFRP structure, but the damage in a CFRP is mainly internal and microscopic, that it is barely visible. Therefore, this study proposes a method of determining impact damage in CFRP via poly(vinylidene fluoride) (PVDF) sensor, which is convenient and has high mechanical and electrical performance. In total, 114 drop impact tests were performed to investigate on impact responses and PVDF signals due to impacts. The test results were analyzed to determine the damage of specimens and signal features, which are relevant to failure mechanisms were extracted from PVDF signals by means of discrete wavelet transform (DWT). Support vector machine (SVM) was used for optimal classification of damage state, and the model using radial basis function (RBF) kernel showed the best performance. The model was validated through a 4-fold cross-validation, and the accuracy was reported to be 92.30%. In conclusion, impact damage in CFRP structures can be effectively determined using the spectral analysis and the machine learning-based classification on PVDF signals.

## 1. Introduction

Carbon fiber reinforced plastics (CFRPs) are widely used in aerospace, military, marine, and automotive industry due to their high specific strength and stiffness [1]. However, CFRPs are easily damaged by transverse loading because fibers in the composite structures are mainly focused on in-plane direction than in the direction normal to plane [2,3]. A CFRP especially suffers low-velocity impacts, such as dropping a tool on the laminate surface during maintenance, bird strikes, and fall of hailstones [3]. The major failure mechanisms caused by a low-velocity impact are matrix cracking, delamination, and fiber failure [4]. These types of damages can reduce the strength of CFRPs up to 30% of that of intact CFRP, and the consequences of impact are likely to be serious, when the damage occurs in civil or military aircraft. [5,6]. However, the damages are barely visible because they are internal, microscopic damage [7]. Therefore, the detection of internal damage induced by impact needs to be carried out, and follow-ups such as repair or replacement of damaged parts should be done. Fiber Bragg grating (FBG) sensors, strain gauges, and ceramic-based piezoelectric sensors have been used to detect impact damages [8]. However, these sensors have problems such as vulnerability to impact damage due to their brittleness and difficulty of integration into a structure [8,9].

The poly(vinylidene fluoride) (PVDF) sensors are being used in the field of structural health monitoring (SHM) due to their high electrical and mechanical performance such as fast electromechanical response, low acoustic impedance, and high impact resistance [8]. Moreover, PVDF sensors are more convenient than conventional sensors as they can be easily manufactured into a variety of shapes and cut or bent to fit into machines in complex shapes [9]. A PVDF sensor can be easily applied to a surface of a test specimen using a bonding tape, and the sensor can easily be removed and reused [10]. Furthermore, they can be embedded into laminates in that a PVDF sensor is a thin-film-type sensor [9,11]. From these results, it can be inferred that a PVDF sensor can be implemented as an effective impact damage detection sensor, since it basically has high impact resistance and can be easily integrated to structures with various geometry.

There have been several studies to detect damages in composite structures using the PVDF sensors. Kim et al. [12] conducted drop impact tests on CFRP specimens and extracted damage signals from vibrations via high-pass filtering, and spectral analysis was done to compare the signals from PVDF and PZT. Bar et al. [13,14] used PVDF sensors as Acoustic Emission (AE) sensors and extracted the AE parameters from the PVDF signals to study the relationship between the parameters and failure mechanisms. Bae et al. [8] studied about the relationship between impact energy and peak voltage of PVDF signals from impacted CFRP structures. However, much less works have been done to analyze the relationships between PVDF signals and impact damage process and mechanisms. This is because in-depth analysis of PVDF signals takes a great effort due to the low sensitivity of PVDF and complexity of the impact damage signals.

Another approach to signal analysis has been to use classification methods based on machine learning algorithms. Most research in this field have focused on analyzing biological signals to predict patient symptoms [15,16,17]. This is due to the complexity of biosignals and the relatively weak correlation between signals and related symptoms. Many researchers have also used machine learning algorithms to analyze damage signals, especially on AE signals or vibration signals, which are effective tools in the field of SHM [18,19,20]. This is mainly due to damping of the signal and effect of boundary conditions or geometry of the specimens that distort signals. The PVDF sensor signals from impact damages in CFRPs are also hard to be analyzed since the damage signals are affected by a variety of failure mechanisms, and signal features can be easily distorted due to damping in the CFRP. Moreover, due to the low sensitivity and damping of signal in the sensor, the signal information can be easily lost when the signals are processed [21]. Therefore, a machine learning algorithm was used to analyze the signal features of the PVDF sensor signals with higher accuracy.

Support vector machine (SVM) is one of the machine learning-based classification algorithms. It has fewer parameters to adjust than other classification algorithms and can classify data with high accuracy even when the training dataset is small [22]. In this study, an impact damage determination model based on SVM was constructed by training the model with PVDF signal features according to the damage state of the impacted specimens. The damage states were determined by analyzing the impact responses of CFRP specimens. Then, discrete wavelet transform (DWT) was used to extract spectral features, which was found to be closely related to failure mechanisms. After that, the damage determination model based on SVM was trained by dataset of signal features labelled as damage state. Finally, 4-fold cross-validation was performed on the models using different kernel functions, for validation of the models and selecting the optimal kernel.

A PVDF sensor, which has a great potential as an impact detection sensor due to its superior mechanical and electrical properties, was not investigated enough due to its low sensitivity and difficulty in signal analysis. In this study, a novel method of determining impact damages in CFRP laminates using a PVDF sensor was proposed. PVDF signals induced by drop impact tests were analyzed in time and frequency domain in comparison with impact response for evaluation of failure mechanisms. The spectral features physically related to failure mechanisms were drawn out using DWT. Subsequently, a SVM algorithm was used to analyze the signal features, which determined the impact damage with the accuracy of 92.30%. In conclusion, the damage states, which was difficult to be analyzed due to the complexity of signals, could be effectively determined by combining spectral analysis and the SVM algorithm.

## 2. Drop Impact Tests

### 2.1. Materials and Experimental Settings

CFRP laminates with the stacking sequence [0°/90°]_13s_ were used. The matrix resin was cured at 125 °C for 90 min. Total thickness of the laminates is 5 mm, descriptions for the mechanical properties of CFRPs provided by the manufacturers (TB Carbon Co, Ltd., Yangsan, Korea) are tabulated in Table 1. In addition, the specimens were trimmed into the dimension of 120 mm × 120 mm using water-jet cutting to prevent mechanical damages in the specimens.

**Figure 1:**
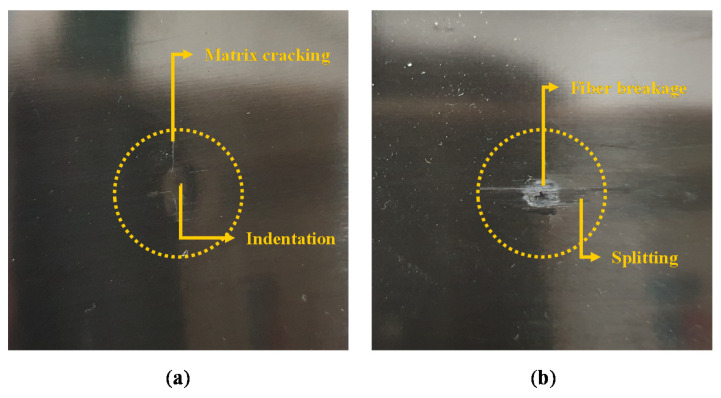
Images of damages in the specimens induced by different impactor shapes: (**a**) a barely visible impact damage induced by a blunt impactor (impactor with R = 4 mm, 12 J impact energy) and (**b**) a visible impact damage induced by sharp impactor (conical impactor, 12 J impact energy).

**Figure 2:**
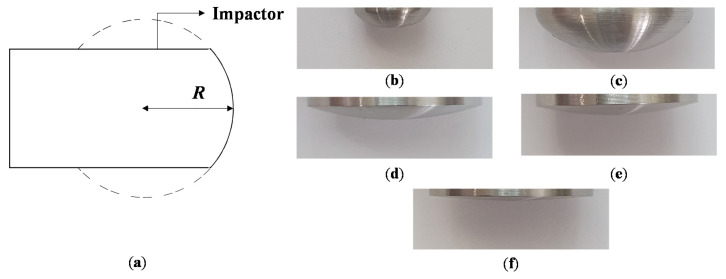
The images of radius of curvature in the nose part of the impactor (R): (**a**) a schematic explanation of *R* of impactor; (**b**–**f**) impactor nose shapes with different R: (**b**) 4 mm, (**c**) 8 mm, (**d**) 16 mm, (**e**) 32 mm, and (**f**) 64 mm.

To analyze the damage in the CFRP specimens and acquire PVDF signals, a PVDF sensor was attached to an impact test specimen using a bonding tape. Drop impact tests were performed under different impact energy levels and radius of curvature in the nose part (R) as Table 2. This is because deformation is mainly dependent on impact force and impactor nose geometry in low velocity impact according to Hertzian contact law, except for the characteristic of the specimen properties (boundary conditions, materials, thickness, layup sequences, fiber orientation, etc.) [23]. However, using specimens with different properties will also affect the propagation of impact signals. Signal characteristics changes will also follow as the signals propagate, and the accuracy of the SVM model will be degraded due to the inconsistency of signal characteristics in the database. In case of impactor with R of 64 mm, drop tests at energy level of 24, 30, and 36 J were also performed because the damage in the specimen occurred at 30 J. In this study, hemispheric impactors were used to induce blunt impact that leaves barely visible damage on surface of CFRP specimen [23]. The example of barely visible impact damage induced by blunt impactor in comparison to the impact by sharp (conical) impactor is shown in Figure 1. The blunt impactor induced matrix cracking and indentation, which were hard to be visually inspected. However, the damage by conical impactor was relatively clearly visible because of the fiber breakage due to the highly localized impact force. Five hemispheric impactors with different R were used. A schematic explanation R and the image of impactor noses are shown in Figure 2.

To analyze the impact damages, the impact load (F), the kinetic energy of the impactor before and after impact, ($E_{i}$), ($E_{a}$) were measured. The absorbed impact energy (E_abs_) and the impact energy ratio (r_abs_) were further calculated as

(1) $$E_{\mathrm{abs}}{\;=\;E}_{i}{-E}_{a}$$

(2) $$r_{\mathrm{abs}}\;=\;\frac{E_{\mathrm{abs}}}{E_{i}}$$

**Figure 3:**
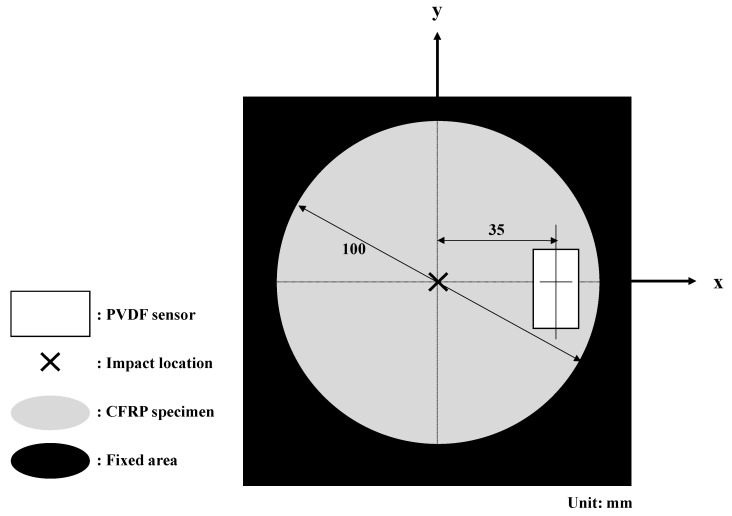
A schematic of the geometry of the test specimens and the location of the poly(vinylidene fluoride) (PVDF) sensor.

**Figure 4:**
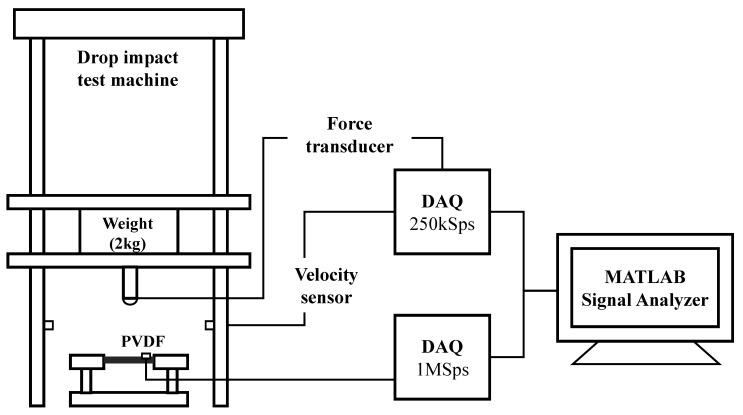
Experimental setup for acquiring PVDF signals and impact response data from drop impact tests.

A PVDF sensor (LDT0-028K) (TE Connectivity, Schaffhausen, Switzerland) was attached 35 mm away from the impact point. The sensor was applied to the surface of a specimen using a double-sided bonding tape and was reused for 20 impact tests because of the reusability of the sensor [10]. New bonding tapes were applied after each test, as the adhesion may be reduced by impacts. Test conditions of the specimens are shown in Figure 3. As shown in Figure 4, the load and PVDF signals from impacts were collected, respectively, through NI USB-6351 (National Instrument, Austin, TX, USA) with sampling rate of 250 kHz and PCI-6133 (National Instrument, Austin, TX, USA) with sampling rate of 1 MHz. The collected data were processed through MATLAB Signal Processing Toolbox 8.3 (Mathworks, MA, USA).

### 2.2. Impact Response

An impact damage in a CFRP consists of various failure mechanisms such as matrix cracking, delamination, and fiber breakages, etc. Delamination is the most critical failure mechanism to strength reduction in composite laminates because it accompanies instantaneous damage [1,2,24,25]. The ultrasonic C-scanning is conventionally used to examine delamination of the damaged specimens. However, C-scanning is time consuming and costly, that it is not adequate for determining damages for the large dataset for machine learning. Failure mechanisms of impact damages could also be effectively analyzed with impact responses of composite laminates [1]. Moreover, the impact response analysis could be immediately done after an impact test. Therefore, impact responses were analyzed to observe the delamination.

**Figure 5:**
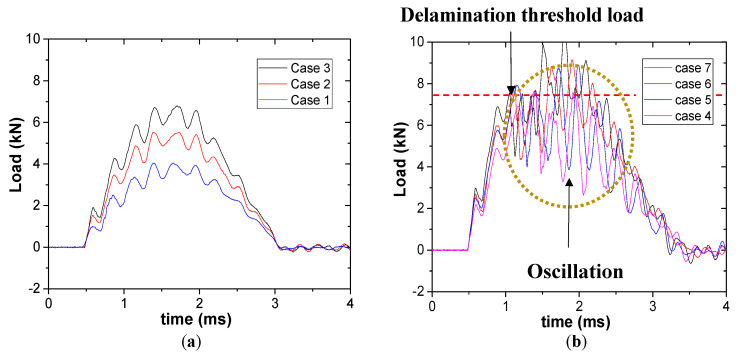
Load-time curve under different impact energy levels: (**a**) 3~9 J and (**b**) 12~15 J.

Figure 5a shows the impact response at the energy levels from 3 to 9 J using impactor with R of 4 mm. However, the load curve shape changes at relatively high impact energy (from 12 to 21 J). Unlike the small load drops that can be observed in Figure 5a, which are from the vibration at the boundary condition, sharp load drops (>2 kN) and following oscillation in load curves were observed in Figure 5b. The load point where the sharp load drop is observed is called the delamination threshold load (DTL) [26]. DTL is the load where bending strain exceeds allowable interlaminar shear strain, followed by rapid growth of internal damage in composite structure [25]. Therefore, the specimens which were loaded beyond their DTL were determined as damaged, and the others which showed elastic behavior upon impact were determined as intact.

**Figure 6:**
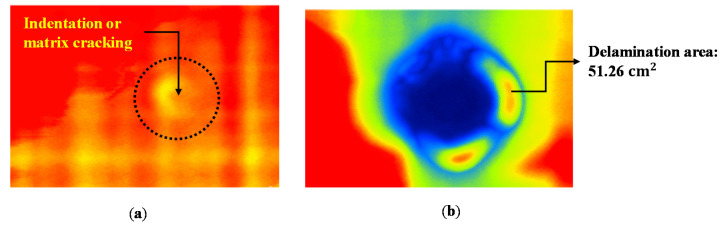
Ultrasonic C-scan images from the impact by R = 4 mm: (**a**) intact specimen (9 J impact) and (**b**) damaged specimen (12 J impact).

The propagation of internal damage by the impact loads exceeding DTL can be observed in Figure 6. The images were obtained using Ez-Scan VII, (Orient NDT, Goyang, Korea) which has analog to digital converter with 200 MHz sampling rate and 10 MHz flat beam immersion transducer. In the intact specimen, a small area of damage at the impact point could be barely seen, which can be inferred as minor matrix cracks or indentation. In the damaged specimen, however, delamination occurred and propagated to the area of 51.26 cm^2^. The damage propagated rapidly can be considered as the impact load exceeded DTL.

**Figure 7:**
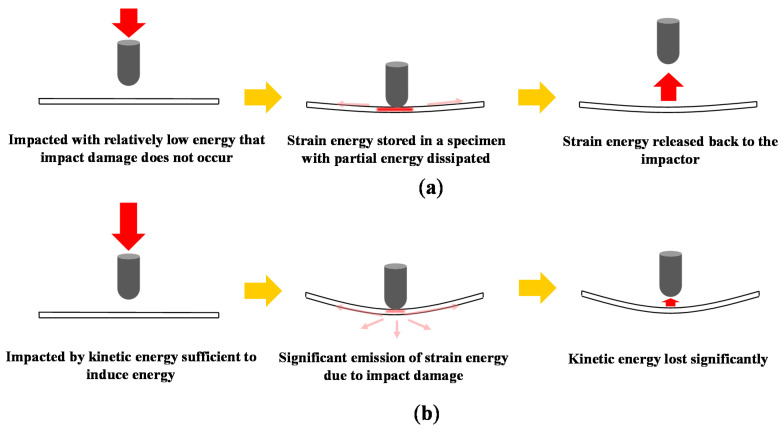
Schematics of impact scenarios: (**a**) the impact scenario where the impacted specimen is intact and (**b**) the impact scenario where damage occurs.

In addition, the damage states of the specimens can be confirmed through a change in the kinetic energy of the impactor. Figure 7 shows a schematic of the kinetic energy loss of impactor that occurs as a result of damage. As the impact load exceeds DTL, the propagation of delamination, additional matrix cracking, and fiber breakages occur, and the kinetic energy of the impactor will drop due to the sudden internal damage leading to strain energy release [2].

## 3. PVDF Signal Analysis

### 3.1. PVDF Signal

**Figure 8:**
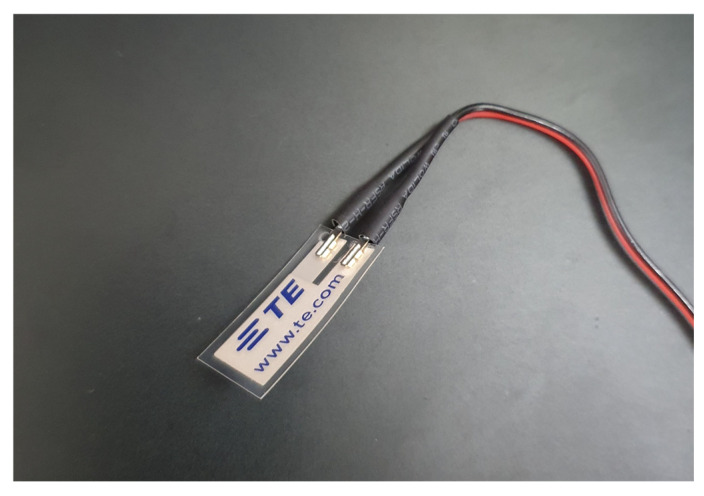
An image of a PVDF sensor (LDT0028K).

A PVDF sensor is a polymer type piezoelectric sensor, which is very thin (0.028 mm) and flexible, that it is capable of being applied to various shapes of structures (Figure 8). A PVDF sensor is capable of measuring stress wave and elastic wave, which is strain energy released upon damage [8]. The stress wave signal, which is below frequency of 20 kHz was filtered out to investigate on elastic waves for damage determination. The reason is that stress waves are mainly dependent on deflection caused by impacts, whereas elastic waves are physically related to the damage, as they are emitted from the damage itself.

**Figure 9:**
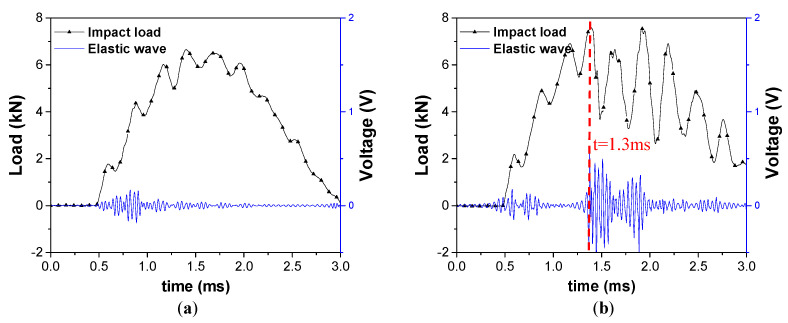
Graphs of elastic waves due to impact load: (**a**) impact energy-9 J, R-4 mm (intact) and (**b**) impact energy-12 J, R-4 mm (damaged).

As an example, elastic waves extracted from PVDF signals induced by impact energy of 9 J (case 3) and 12 J (case 4) were analyzed (Figure 9). The elastic waves, which are emitted at 0.5 ms in both Figure 9a,b can be assumed as signals from matrix cracking, which occurs at low impact energy. At 1.3 ms of Figure 9b, the impact load reaches DTL; therefore, the matrix and fiber damage, and delamination occur, emitting elastic waves with high energy.

The damages could be analyzed by comparing the elastic waves with the load curves. However, the waveform of the damage signal is not reliable due to the high damping coefficient of the CFRP [27]. In contrast, the spectral features are known to be reliable features. One-dimensional spectral features such as the peak frequency and center frequency of the elastic waves are effectively used to analyze failure mechanisms [28,29]. However, when it comes to impact damage, elastic waves from damage consist of signals from a variety of failure mechanisms. Therefore, DWT, one of the time–frequency domain analysis methods was used to analyze the spectral features over all frequency ranges of the elastic waves.

### 3.2. Wavelet Transform

Wavelet transform (WT) is a type of the time–frequency analysis method that decomposes a signal based on mother wavelets. Mother wavelets are finite waves unlike sinusoidal waves, which are used in Fourier transform. For these reasons, WT is adequate for decomposing the PVDF signals from impact damages since nonstationary elastic wave from various failure mechanisms are combined when impact damage occurs [30].

The basic WT, continuous wavelet transform (CWT), decomposes a signal in time and frequency domain, and the decomposition of the signal X(t) based on mother wavelet $\varphi^{*}$ can be defined as

(3) $$\mathrm{CWT}(a,b)\;=\;\frac{1}{{\left|a\right|}^{0.5}}\int_{-\infty }^{+\infty }X\left(t\right)\varphi^{*}\left(\frac{t\;-\;b}{a}\right)\mathrm{dt}$$

where a is the scaling parameter, used to scale the amplitude of wavelet signals, and b is the translation parameter, which shifts the signal in time domain. In this study, DWT was used and the DWT decomposing signal X(t) is defined as

(4) $$\mathrm{DWT}(m,n)\;=\;\frac{1}{{\left|2^{m}\right|}^{0.5}}\int_{-\infty }^{+\infty }X\left(t\right)\varphi^{*}\left(\frac{{t-2}^{m}n}{2^{m}}\right)\mathrm{dt}$$

where m and n are integers that substitute scaling parameter and translation parameter as $2^{m}$ and $2^{m}n$, respectively. Therefore, DWT takes much less computation time DWT, while preserving the information in signal [31,32].

**Figure 10:**
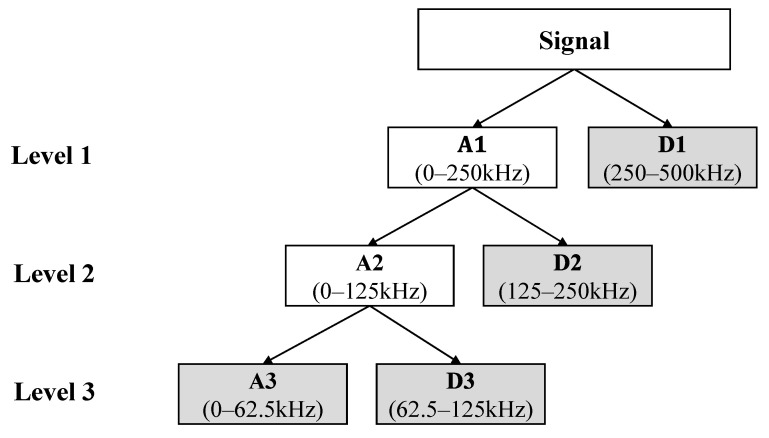
A decomposition tree structure of three-level discrete wavelet transform (DWT) decomposition and result of decomposition (A3 and D1–D3).

The result of DWT is an approximation coefficient (A1) and a detailed coefficient (D1) each. The approximation coefficient is a signal with lower frequency range, and the detailed coefficient is the signal with higher frequency range. The maximum frequency is 500 kHz based on Nyquist criterion. Therefore, the frequency range of the approximation coefficient is 0 kHz–250 kHz, and the frequency range of the detailed coefficient is 250–500 kHz. The approximation coefficient can be decomposed into another approximation coefficient and detailed coefficient again. This means lower frequency signals can be decomposed until the targeted frequency bands are acquired. This is explained in Figure 10, which is the example of three-level DWT.

In this study, PVDF signals were decomposed to three levels using a Daubechies wavelet. The Daubechies wavelets have been used for decomposing elastic waves in other relevant studies, because the Daubechies wavelets are efficient for analyzing transient features of the signals [18,30,32,33,34,35]. Daubechies wavelet with four vanishing moments were used, since it was effective in decomposing damage signals in relevant studies [30,35].

### 3.3. Feature Extraction

**Figure 11:**
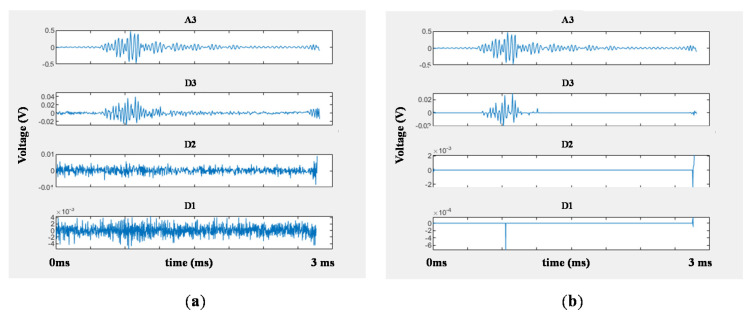
The decomposed signals from 9 J impact, with R of 4 mm: (**a**) the signal before denoising and (**b**) the signal denoised using soft thresholding.

Prior to analyzing decomposed signals, the signals were denoised by means of DWT denoising, which is a technique of reducing the noise of every DWT coefficients. The signals were denoised using soft thresholding, since hard thresholding technique can leave some traces of noise [30]. The example of the reduction in noise is shown in Figure 11.

**Figure 12:**
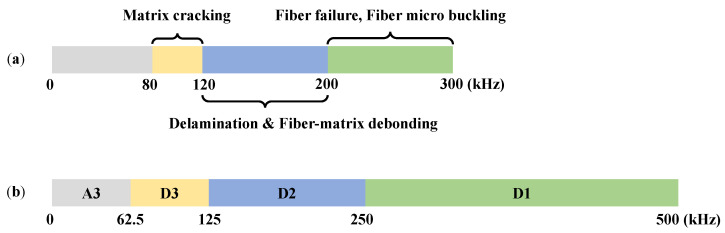
Frequency contents of elastic wave according to: (**a**) failure mechanisms in carbon fiber reinforced plastic (CFRP) cross-ply laminates by Arumugam et al. [36] and (**b**) coefficients of a PVDF sensor signal acquired by three-level DWT.

Spectral features of a signal are reliable since the frequency of the signal is only slightly affected by the damping and boundary conditions compared to the time domain features. Figure 12a shows the peak frequency of the elastic wave according to the failure mechanism defined in the study by Arumugam et al. [36], which was obtained by analyzing the elastic wave signal from quasi-static bending tests and impact tests on CFRP cross-ply laminates. Figure 11b is the frequency band of the coefficients from DWT. The detailed coefficients closely match with the frequency bands of the matrix cracking, delamination and matrix debonding, and fiber failure and fiber microbuckling. Therefore, DWT was performed to level 3, since the approximation coefficient does not need to be further decomposed.

**Figure 13:**
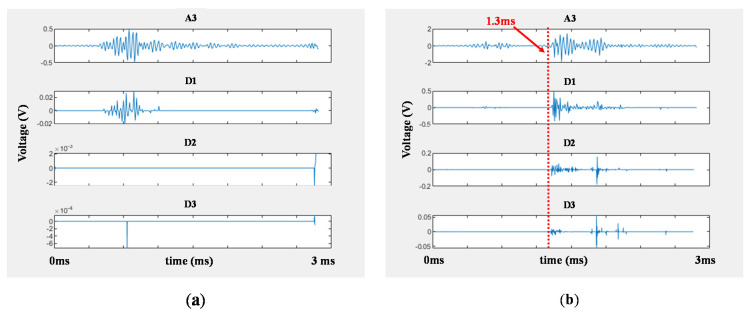
DWT coefficients of PVDF sensor signal of different damage state: (**a**) impact energy 9 J (intact) (**b**) impact energy 12 J (damaged).

The D3 signal, which corresponds to the matrix cracking, is dominant before the delamination initiates (amplitude 0.022 V), as can be seen in Figure 13a. In Figure 13b, the impact load exceeds DTL at 1.3 ms. After a strong signal corresponding to matrix cracking, (amplitude 0.498 V), delamination and fiber breakage follows (D1 and D2). These physical processes match well with previous studies showing that the DWT coefficients of the PVDF sensor signals represent failure mechanisms properly [26,37]

The energy ratios of DWT coefficients were extracted to construct feature dataset for training damage determination model. The reason is that energy ratios of frequency contents not only can be used for quantitative representation of failure mechanisms [33,36,38] but also are reliable features, which are less effected by the boundary conditions and impact location [39]. The name and frequency range of the extracted features are organized in Table 3. Calculation of signal energy E_k_ and energy ratio Er_k_, where X and t are signal voltage and time, respectively, is defined as follows.

(5) $$E_{k}\;=\;\int^{\;}{|X}_{k}\left(t\right){|}^{2}\mathrm{dt}\;\left(\mathrm{for}\;k\;=\;1,2,3,4\right)$$

(6) $${\mathrm{Er}}_{k}\;=\;\frac{E_{k}}{E_{1}+E_{2}+E_{3}+E_{4}\;}\;\left(\mathrm{for}\;k\;=\;1,2,3,4\right)$$

## 4. Support Vector Machine

### 4.1. Support Vector Machine Algorithm

**Figure 14:**
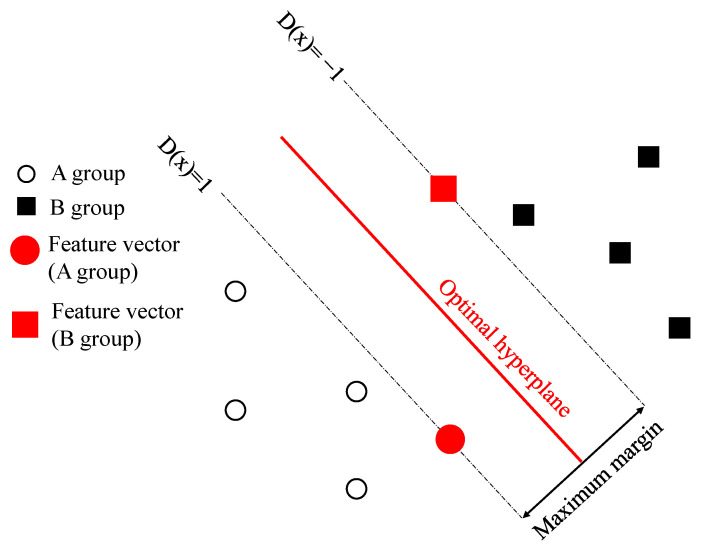
A schematic explanation of relationships between hyperplane, margin, decision function D(**x**), and feature vectors.

SVM is a supervised classification method based on optimization theories. SVM is trained to maximize the margin between the classified groups. The optimal boundary that maximizes the margin is called a hyperplane, which is determined by support vectors. Support vectors are feature vectors that are closest to the classification boundary [40]. SVM techniques have good generalization capability for small-sample cases of classification [41] and are utilized in many fields, including fault and damage detection [18,22,34,38,41]. A schematic of SVM is shown in Figure 14 to visualize the relationships between feature vectors, decision function D(x), margin, and hyperplane, before further descriptions of SVM algorithm [40].

SVM used to determine which group of n-dimensional feature vectors x_k_ is classified into A or B. The label y_k_ value according to the feature vector is expressed as follows.

(7) $$\mathrm{For}\;\left(x_{1}{,\;y}_{1}\right),\left(x_{2}{,\;y}_{2}\right),\left(x_{3}{,\;y}_{3}\right),\;\ldots ,\left(x_{p}{,\;y}_{p}\right)\;{\mathrm{If}\;x}_{k}\;\in {\;A,\;y}_{k}\;=\;1\;\;{\mathrm{If}\;x}_{k}\;\in {\;B,\;y}_{k}\;=\;-1$$

The support vector machine algorithm determines the parameters of the determination function through learning of these feature vectors, and the process follows the rules,

(8) $${D(x}_{k}{)\;=\;w\times x}_{k}+b$$

where w and b are the adjustable parameters of the decision function.

(9) $${\mathrm{If}\;x}_{k}\in A,\;D\left(x_{k}\right)\;\geq \;1\;{\mathrm{If}\;x}_{k}\in B,\;D\left(x_{k}\right)\;\leq \;-1\;$$

The plane that is nearest to hyperplane can be described as Equation (10)

(10) $$y_{k}D\left(x_{k}\right)\;-1\;=\;0$$

The two feature vectors x_A_ and x_B_ that satisfies Equation (10) are support vectors, and the margin M is the distance between the planes $D\left(x_{A}\right)\;=\;1$ and $D\left(x_{B}\right)\;=\;-1$

(11) $$M\;=\;\frac{y_{A}D\left(x_{A}\right)}{\left|\left|w\right|\right|}-\frac{y_{B}D\left(x_{B}\right)}{\left|\left|w\right|\right|}\;=\;\frac{2}{\left|\left|w\right|\right|}\;$$

The margin M can be expressed using parameter was Equation (11). The SVM algorithm is an optimization problem of maximizing the margin. Maximizing the margin can be transformed into minimizing problem as follows.

(12) $$\max\left(M\right)\;=\;\max\frac{2}{\left|\left|w\right|\right|\;}\to \;\min\left|\left|w\right|\right|\to \;\min{\left|\left|w\right|\right|}^{2}$$

Equation (12) can be expressed as Equation (13) using Lagrangian where α is a Lagrange multiplier,

(13) $$L\left(w,b,\alpha \right)\;=\;\frac{1}{2}{\left|\left|w\right|\right|}^{2}-\overset{p}{\underset{k\;=\;1}{\sum }}\alpha_{k}{[y}_{k}D\left(x_{k}\right)-1]$$

which satisfies conditions

(14) $$\alpha_{k}\;\geq {\;0,\;k\;=\;1,2,3,\ldots ,p,\;\alpha }_{k}\left[y_{k}D\left(x_{k}\right)-1\right]\;=\;0$$

The minimum value of L could be calculated by differentiation

(15) $$\frac{\partial L}{\partial w}{\;=\;w}^{*}-\overset{p}{\underset{k\;=\;1}{\sum }}\alpha_{k}^{*}y_{k}x_{k}\;=\;0$$

Hence,

(16) $$w^{*}\;=\;\overset{p}{\underset{k\;=\;1}{\sum }}\alpha_{k}^{*}y_{k}x_{k}$$

The optimal parameter w^*^ is given as Equation (16) and b can also be calculated by Equation (14). Finally, equation for hyperplane can be achieved by calculating the plane in the middle of D(x_A_) and D(x_B_).

### 4.2. Kernel Function

**Figure 15:**
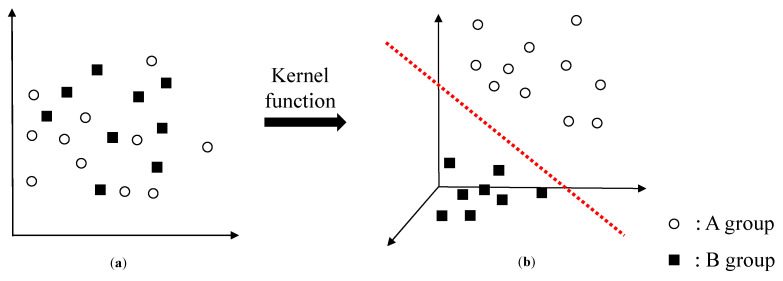
Schematic diagrams showing: (**a**) feature vectors which are not linearly separable and (**b**) feature vectors mapped into new feature space via kernel function.

The SVM algorithm described earlier is not adequate for classifying the dataset in Figure 15a into A or B, since it is only effective in linearly separable data. In this case, feature vectors are mapped onto a new feature space as Figure 9b, using kernel function. The kernel function K is used to convert dot product of feature vectors.

(17) $$x_{k}^{T}x_{m}\;\to {\;K(x}_{k}{,x}_{m}).$$

The representative kernels are Equation (18) linear kernel, Equation (19) sigmoid kernel, Equation (20) polynomial kernel, and Equation (21) RBF (radial basis function) kernel.

(18) $$K\left(x,y\right){\;=\;x}^{T}\cdot y$$

(19) $$K\left(x,y\right){\;=\;\tan h(\mathrm{ax}}^{T}\cdot y+b)$$

(20) $$K\left(x,y\right)\;=\;\;{\left(x^{T}\cdot y+c\right)}^{d}$$

(21) $$K\left(x,y\right)\;=\;\exp{\left(-\gamma \left|\left|x^{T}\cdot y\right|\right|\right)}^{2}$$

### 4.3. SVM-Based Impact Damage Determination Model

**Figure 16:**
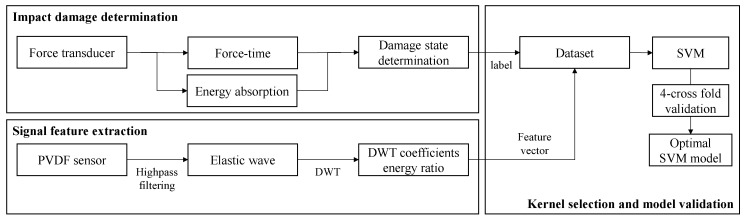
Process of constructing the impact damage determination model via support vector machine (SVM) algorithm.

The process of determining damage states based on the SVM algorithm is shown in Figure 16. To train the damage determination model, the impact responses were analyzed to determine the damage state of the specimens. The results were used to label the feature vectors x_k_, which are the energy ratios of DWT coefficients.

(22) $$x_{k}\;=\;\left({\mathrm{Er}}_{1}{,\;\mathrm{Er}}_{2}{,\;\mathrm{Er}}_{3}{,\;\mathrm{Er}}_{4}\right)\;{\mathrm{If}\;x}_{k}\;\in {\;\mathrm{Damaged},\;y}_{k}\;=\;1\;\;{\mathrm{If}\;x}_{k}\;\in {\;\mathrm{Undamaged},\;y}_{k}\;=\;-1$$

When a new feature vector is input to the trained SVM model, the damage state of the specimen is determined by the spatial relation between the hyperplane and the feature vector; whether it is on one side of the divided space or the other.

Furthermore, 15 additional impact tests were performed at energy levels where damage initiated, in that better quality of hyperplane can be generated by adding features close to the boundary of the classes (Table 4).

### 4.4. Model Evaluation

**Figure 17:**
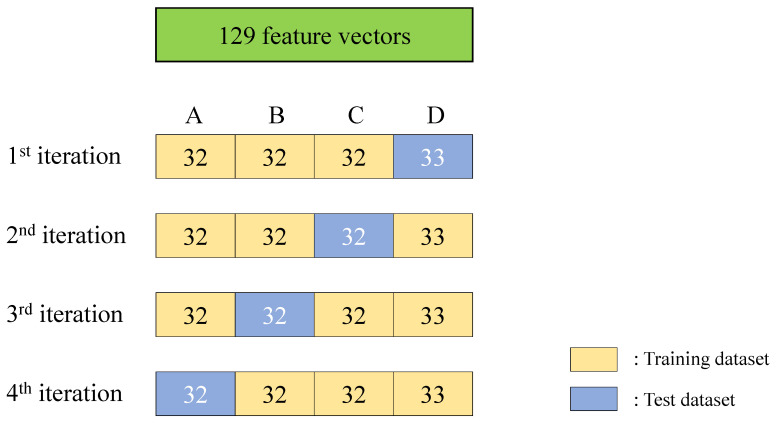
A schematic of 4-fold cross-validation for dataset with 129 feature vectors.

The performance of the SVM depends on the choice of kernel function, and there are no definite rules of selecting proper kernel function [42]. Therefore, we trained and tested performance of models using different kernel functions to choose the best one. To validate SVM models using different kernels, we used 4-fold cross-validation, which is effective in preventing overfitting, and also evaluates the model when the dataset is small [43]. A schematic of 4-fold cross-validation for total 129 feature vectors is shown in Figure 17. “Training data” is the dataset used to construct classification model, and “test data” is the data which is input to the trained model to validate whether the model predicted the label of the data correctly or not. The data is divided into four groups of A, B, C, and D randomly. The components of the confusion matrix, which is descripted in Table 5 were used to compute the accuracy, precision, recall, and average precision (AP) for each iteration. The performance values for four iterations were averaged, so that every data is used for validating the performance, and overfitting due to the lack of test data can be prevented.

- Accuracy is the most intuitive indicator of the classification performance of a model, and it is the probability that the model will correctly judge whether a specimen is damaged or not.
  (23) $$\mathrm{Accuracy}\;=\;\frac{\mathrm{TP}+\mathrm{TN}}{\mathrm{TP}+\mathrm{FP}+\mathrm{NP}+\mathrm{TN}}$$
- Precision is the ratio of features that are actually “damaged,” among the features the model classified as damaged.
  (24) $$\mathrm{Precision}\;=\;\frac{\mathrm{TP}}{\mathrm{TP}+\mathrm{FP}}$$
- Recall is the ratio of features predicted as “damaged” among features that are actually “damaged”.
  (25) $$\mathrm{Recall}\;=\;\frac{\mathrm{TP}}{\mathrm{TP}+\mathrm{FN}}$$
- AP is frequently used over precision or recall for performance validation, which is the measure of mean precision at a set of equally spaced recall levels [0, 0.1, …., 1]:
  (26) $$\mathrm{AP}\;=\;\frac{1}{11}\overset{}{\underset{r\in \{0,0.1,\ldots ,1\}}{\sum }}p_{\mathrm{interp}}\left(r\right),$$
  where r is recall value and p_interp_ is the interpolated line of maximum precision at each recall level r. Further descriptions are given in [44].

## 5. Results and Discussion

### 5.1. Damage State Determination

In this study, a total of 114 drop impact tests were performed to analyze the impact damage in CFRPs. The impact load and energy absorption ratio were evaluated to determine impact damage by each impact tests. To train machine learning-based classification model for damage determination, the damage determination results, which will be used to label feature vectors, must be exact and precise. Otherwise, the SVM model cannot be considered valid, as the label information is not credible. The damage of the specimens was determined as “intact” or “damaged,” depending on the occurrence of delamination. This is because delamination accompanies instantaneous damage and resulting strength loss [2,25].

**Figure 18:**
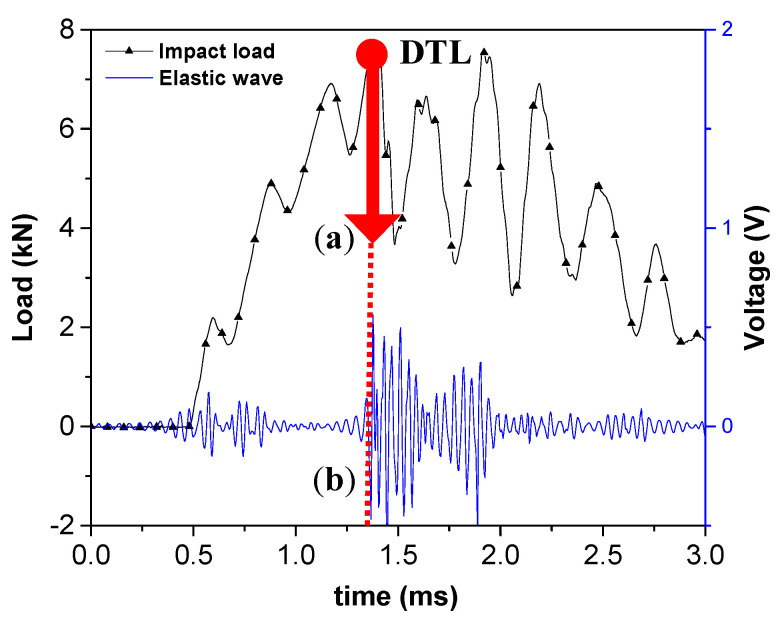
Determination of delamination threshold load (DTL) point based on: (**a**) a sudden load drop and (**b**) emission of elastic waves.

**Figure 19:**
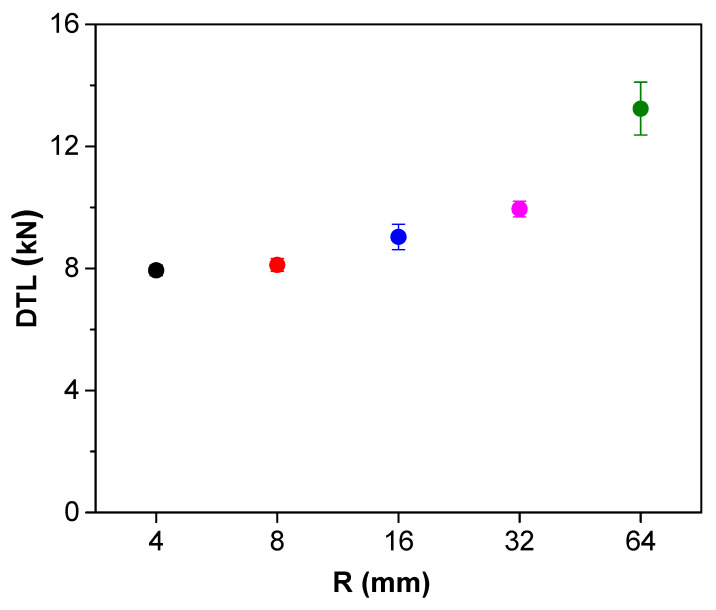
DTL according to R of impactors as a test result of 114 drop impact tests.

The description of determining the DTL value is given in Figure 18. The elastic waves were emitted at 1.3 ms, and the load dropped about 4 kN at the same time. It can be inferred that strong elastic waves were emitted as delamination occurred, and load also dropped as the local stiffness at the impact point was degraded due to the damage. Therefore, the load point where significant load drop and emission of elastic waves occur at the same time is determined as DTL. DTL according to five different impactor R are shown in Figure 19. The results reported that DTL converges to a certain value when the R of impactor is constant, and that value increases with R. This is due to change of force distribution; impactors with larger R causes less localized strains due to larger contact area. In that reason, it requires higher impact energy and impact force for damage initiation. It can be inferred that impactor of different nose shapes induce different types of damages.

**Figure 20:**
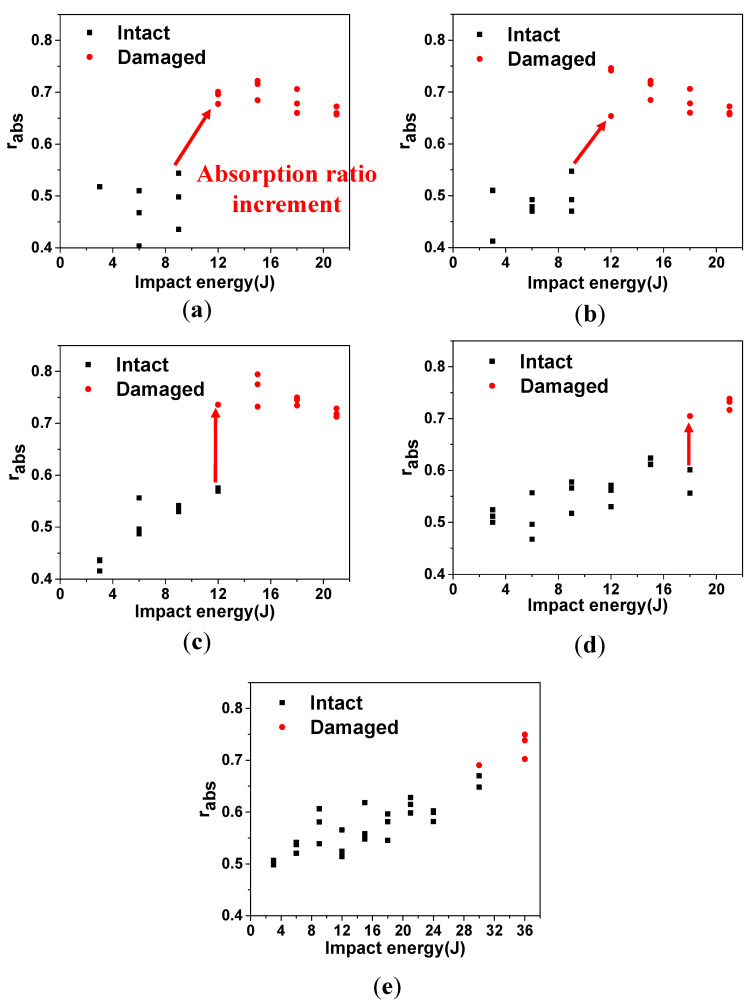
Impact energy absorption ratio increment of different R: (**a**) 4 mm, (**b**) 8 mm, (**c**) 16 mm, (**d**) 32 mm, and (**e**) 64 mm.

In addition, impact energy absorption ratios were analyzed as shown in Figure 20 to confirm the occurrence of significant impact damage when impact load exceeds DTL. The energy absorption ratio increments were significant: 24.6%, 19.4%, 27.8%, and 15.3%. These values correspond to the increments marked with the pointers in case of Figure 20a–d. The increment values of absorption ratio are scattered because the impact damage in composite laminates does not occur by similar extent, even when the test is performed under the same impact conditions [26]. Although exact severity of the damages cannot be achieved, identical transition of the energy absorption ratio in case of Figure 20a–d sufficiently explain that significant damage occurred as the impact load exceeded DTL. However, in case Figure 19e, the damage absorption ratio did not increase as much as it did in impactors with R of 4–32 mm (3%), since impact by R of 64 mm required higher impact energy for damage to initiate compared to impactors with R of 4–32 mm, and the higher impact energy and force for damage initiation increases the damage at locations away from impact site [23]. This results in less damage at impacted location, which means less stiffness decrease and less impact energy absorption of impactor.

Moreover, signal energies according to damage state were examined for quantitative comparison intensity of signals according to damage state. The significant increase in signal energy upon damage initiation could be observed in every impact condition as shown in Table 6. This result validates the dramatic growth of damage as delamination proceeds. In addition, the damage state classification result on 64 mm R impact could be considered valid despite the small increase in energy absorption ratio since signal energy increased by 8.32 times as delamination occurred.

In conclusion, it is reasonable to classify impact damage in CFRP based on the initiation of delamination, in that it accompanies rapid growth of damage. This result was validated by load curve, energy absorption analysis, and PVDF signal analysis.

### 5.2. Signal Dataset Construction and Analysis

In total, 129 (114 tests + 15 additional tests) of energy ratio feature vectors were labelled with damage states, which were determined by impact response analysis. The descriptive statistic of total 129 feature vectors (74 I (intact) + 55 D (damaged)) is tabulated in Table 7. Each feature was normalized to be in scale between 0 and 1, for better comparison of the values. As shown in the results, the means of features Er_2_, Er_3_, and Er_4_, which correspond to ratio of failure mechanisms in the specimens, increased by 8.3, 38, and 82 times each, respectively. This significant change of features is due to instant damage growth as delamination occurred.

**Figure 21:**
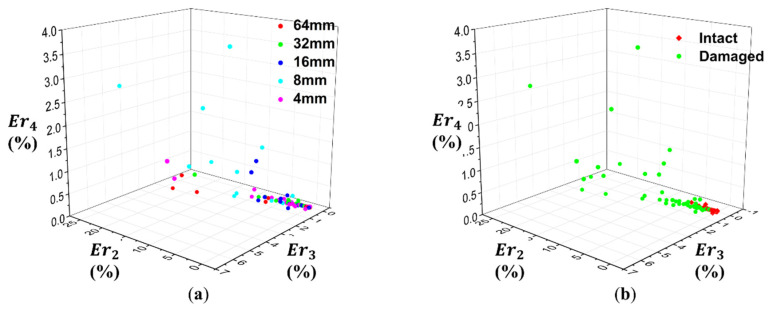
Feature dataset visualized in feature space according to: (**a**) R of impactor and (**b**) damage state.

The feature dataset was visualized in Figure 21, where 3 coordinates correspond to energy ratio of detailed coefficients, Er_2_, Er_3_, and Er_4_. It can be considered that the distribution of feature value depends upon damage state of the specimens and shows little correlation with impact condition. This is because the PVDF sensor was unable to capture the change of failure mechanisms by the effect of nose shapes. However, the change of failure mechanism ratio according to the damage state could be measured, because of the significant damage due to delamination.

### 5.3. SVM Model Validation

Energy ratios of frequency bands were proven to be effective features for determining damage state. However, some data could not be clearly differentiated by damage states due to low sensitivity and high damping of PVDF sensors. Therefore, SVM was used to make a clear boundary that classifies feature vectors for decisive damage determination.

Damage determination results used to train the SVM model was acquired as two states, based on the physical process where internal damage in composite laminates starts to grow. Moreover, the damage determination result was validated by analyzing impact energy absorption ratio and signal energies. Therefore, the performance of the classifier presented in Table 8 is valid. The performance validation result of 4-fold cross-validation over 4 representative kernel functions is summarized in Table 8.

The model using sigmoid kernel failed to determine every damaged state, and can be considered that sigmoid kernel is not appropriate in this model. The model using polynomial function had the longest training time despite its lower accuracy compared to the model using the linear kernel. The model using linear kernel, which is the simplest form of SVM, was found to be compliant, with accuracy of 90.79% and the shortest training time. The model using RBF kernel showed the best performance, with accuracy of 92.30%, and was also found to have highest AP value of 0.89. Therefore, it can be considered that the model using RBF kernel is most adequate for determining impact damage by analyzing PVDF signal data.

To sum up the results, the damage state was determined by impact response, and SVM models were trained with signal features to predict damage state of the impacted specimen. The accuracy of the model using RBF polynomial kernel was confirmed to be 92.30%. This can be considered a valid result, since this accuracy is higher compared to similar research, despite lack of data [45,46]. From these results, the SVM algorithm-based impact damage state classification method presented in this study is reasonable.

## 6. Conclusions

In this article, PVDF signals induced by impact damages in CFRP specimens were measured, and the determination of damages was performed based on DWT and a SVM algorithm. The PVDF sensor signals were analyzed in time–frequency domain in accordance with impact process. Moreover, by employing a machine learning algorithm, damage state of the specimens could be classified optimally. Consequently, the impacts which barely have any effect on strength of CFRP structures, which could be sorted out quantitatively. The conclusions of this study are as follows.

1. The impact responses of CFRP cross-ply laminates specimens were analyzed to determine the damage state in test specimens. The specimens loaded over their DTL were considered to include delamination, and the resulting delamination could be observed using C-scan. The specimens were classified into “damaged” and “intact,” based on the occurrence of delamination, where rapid damage growth in CFRP initiates. Energy absorption ratios increased by at least 15% to as much as 27% higher as damage occurred, indicating the validity of damage classification. The energy absorption ratio increased by only 3% in case of impact damage induced by impactor with R of 64 mm. However, this is due to the less localized force and damage. The damage classification result can still be considered valid since PVDF signal energy increased by 8.32 times according to the damage in the specimens, when compared to the signal energy from intact specimen, implying that the significant damage was developed in the specimens.
2. PVDF signals were divided in frequency domain by means of three level DWT. Each frequency band of detailed coefficients (D1, D2, and D3) were found to represent failure mechanisms of CFRP through comprehensive analysis with load curve. For quantitative evaluation of failure mechanisms, energy ratio from each DWT coefficients were extracted as Er_1_–Er_4._, which represent reverberation and frequency, matrix cracking, delamination and debonding, and fiber damage in CFRP, respectively. The coefficients Er_2_, Er_3_, and Er_4_ significantly increased by 8.3, 38, and 82 times each, respectively, as delamination occurred.
3. A SVM algorithm was utilized to create an optimal border to classify feature vectors for determining damage state clearly. Of all the models using different kernel functions, the model using RBF kernel showed the highest accuracy and AP of 92.30% and 0.89, respectively. This is a reasonable reliability, which are compliant compared to the accuracy of previous relevant studies. Based on these results, determining the impact damage in CFRP using SVM method can be considered effective.
4. The novel methodology of determining the damage state of CFRPs based on PVDF signals was proposed. This work emphasizes on the damage state determination despite of the low sensitivity of sensor and complexity of the signals by combining spectral analysis and a SVM algorithm. A PVDF sensor could not be used for accurate impact damage determination sensor despite its high impact resistance and convenience, because of its low sensitivity and complex nature of the impact damage signal. By studying on the relationships between impact damage and process and PVDF signal characteristics, significant difference in DWT coefficients upon impact damage was identified. Moreover, SVM was used for definite determination of impact damages since some data could not be clearly separated due to the low sensitivity of the sensors. As a result, the damage could be determined with high accuracy. This method can be applied for monitoring CFRP structures, since PVDF sensors can fit into various structures compared to conventional sensors. Moreover, because this method utilizes machine learning for damage determination, impact damage in composite laminates can be automatically determined. Therefore, it would save a lot of time and efforts for analyzing and detecting damages.

## Figures and Tables

**Table 1:** The mechanical properties of carbon fiber reinforced plastics (CFRPs).

Tensile Strength ^1^ (0°) (MPa)	Tensile Strength ^1^ (90°) (MPa)	Interlaminar Shear Strength ^2^ (MPa)	Density (g/cm^3^)
2520	134	88	1.59

Acquired by ASTM D ^1^ 3039 and ASTM D 2344 ^2^ on a UD laminate.

**Table 2:** Impact test cases according to the impact energy and *R.*

Case Number	Impact Energy (J)	*R* (mm)
1	3	4
2	6	
3	9	
4	12	
5	15	
6	18	
7	21	
8	3	8
9	6	
10	9	
11	12	
12	15	
13	18	
14	21	
15	3	16
16	6	
17	9	
18	12	
19	15	
20	18	
21	21	
22	3	32
23	6	
24	9	
25	12	
26	15	
27	18	
28	21	
29	3	64
30	6	
31	9	
32	12	
33	15	
34	18	
35	21	
36	24	
37	30	
38	36	

**Table 3:** Energy ratio of three-level discrete wavelet transform (DWT) coefficients, its frequency range, and related failure mechanisms.

Energy Ratio Feature	Frequency Range (kHz)	Related Failure Mechanisms
Er_1_	0.00–62.50 (A3)	– (Friction and reverberation)
Er_2_	62.50–125 (D3)	Matrix cracking
Er_3_	125–250 (D2)	Delamination Matrix debonding
Er_4_	250–500 (D1)	Fiber failure Fiber microbuckling

**Table 4:** Additional drop impact tests at the damage initiating energy level.

Additional Case Number	Damage Initiation Impact Energy Range	R (mm)	Impact Energy (J)
1	9–12	4	10
2			11
3			12
4	9–12	8	10
5			11
6			12
7	15–18	16	15
8			16
9			17
10	18–21	32	18
11			19
12			20
13	30–36	64	30
14			32
15			34

**Table 5:** Confusion matrix of impact damage determination model.

	Damaged (Predicted)	Intact (Predicted)
**Damaged** **(real)**	True positive (TP)	False negative (FN)
**Intact** **(real)**	False positive (FP)	True negative (TN)

**Table 6:** Signal energies of elastic wave according to damage state I (intact) and D (damaged).

*R* (mm)	4		8		16		32		64	
Damage state	I	D	I	D	I	D	I	D	I	D
Impact energy(J)	9	12	9	12	12	15	18	21	30	36
Signal energy $\left(V^{2}\cdot s\right){\;\times \;10}^{-6}$	0.9842	19.341	1.2468	25.530	1.1853	18.899	2.0235	24.549	3.3408	27.807

**Table 7:** Statistic descriptions of normalized energy ratio of Er_1~4_ according to damage state.

Feature	Mean		Standard Deviation	
Damage state	I	D	I	D
Er_1_	0.9731	0.7337	0.03145	0.2076
Er_2_	0.0337	0.2799	0.03946	0.2123
Er_3_	0.0054	0.2063	0.01176	0.2482
Er_4_	0.0025	0.2050	0.00678	0.2050

**Table 8:** Performance of classifiers using different kernel functions.

Kernel Function	Training Time (s)	Accuracy (%)	Precision (%)	Recall (%)	AP
Linear	0.0007	90.79	89.65	88.87	0.8434
Polynomial	0.13	87.56	91.77	77.61	0.8048
RBF	0.003	92.30	97.91	83.51	0.8867
Sigmoid	0.00025	58.14	0	0	0.4285
